# 

**Published:** 1907-06-29

**Authors:** 


### BOOKS RECEIVED.

Baillière, Tindall, and Cox.

"Dictionary of Medical Diagnosis." By H. L. McKisack, M.D.

Ash and Co., Ltd.

"Guy's Hospital Nursing Guide," 1907.

John Murray.

"The Licensed Trade." By E. A. Pratt.

H. J. Glaisher.

"Hay Fever, or Hay Asthma: Its Causes, Diagnosis, and Treatment." By Wm. Lloyd.

THE HOSPITAL *June* 29, 1907.

*Name* ..........................................................................

*Address* ..........................................................................

..........................................................................

This Coupon must accompany manuscript or contributions intended for The Hospital.

